# Professional identity formation within Longitudinal Integrated Clerkships: a scoping review protocol

**DOI:** 10.1186/s13643-020-01422-6

**Published:** 2020-07-24

**Authors:** Megan El Brown, Paul Whybrow, Gavin Kirwan, Gabrielle M. Finn

**Affiliations:** 1 Health Professions Education Unit, Hull York Medical School, University of York, York, UK; 2 Academy for Primary Care, Hull York Medical School, University of Hull, Hull, UK; 3 York, UK

**Keywords:** Professional identity, Identity, Longitudinal Integrated Clerkship, Scoping review, Medical student

## Abstract

**Background:**

Professional identity development is an area of contemporary interest within medical education. It can be defined as ‘the foundational process one experiences during the transformation from lay person to physician’. In order for this transformation to occur, medical values and principles are internalised. A robust professional identity is key to confident practice as a medical professional. As such, research regarding what works to encourage identity development is popular. New models of educational delivery, such as the increasingly popular Longitudinal Integrated Clerkship model (LICs), present an interesting opportunity to investigate impact on identity. As no previous literature reviews focus on identity development within LICs, it is unclear what is already known about their impact. Therefore, a scoping review synthesising current knowledge and mapping areas for future research is necessary.

**Methods:**

Arksey and O’Malley’s scoping review steps will be used as a methodological framework. MEDLINE, EMBASE, PubMed, Web of Knowledge, ERIC, PsychINFO, Google Scholar, JSTOR, Scopus, and Web of science will be searched (from inception onwards). We will include single studies of any design (e.g. quantitative and qualitative) and reviews examining professional identity within Longitudinal Integrated Clerkships involving health profession students. Two reviewers will complete all screening and data abstraction independently. Deductive coding will be presented as a quantitative textual meta-analysis. Inductive coding will be presented in narrative format.

**Discussion:**

This scoping review will explore professional identity formation within LICs, evaluating any known impact of the educational model and mapping the ways in which identity within LICs has been researched. Mapping of current knowledge should highlight whether LICs as an educational model can influence professional identity development and outline gaps in what is known about their impact to date. Theory used in LIC-based identity research will also be mapped, in order to summarise the main theoretical orientations of research to date. It is anticipated that through such evidence synthesis, directions for future research will become clear.

**Systematic review registration:**

Open Science Framework: osf.io/hk83p

## Background

Professional identity is increasingly studied within medical education, particularly amongst medical students and physicians, where its creation may be defined as ‘the foundational process one experiences during the transformation from lay person to physician’ [1]. Developing a healthcare professional’s identity requires internalisation of ‘core values… moral principles and self-awareness’ [1] and allows professionals to ‘practice with confidence and professionalism’ [2]. Some have even suggested robust identity development could prove protective against medical burnout [3]. It is no surprise, therefore, that institutions and educators are increasingly looking to explicitly encourage professional identity development within healthcare education. As the best way to encourage identity development within medical students remains unclear, further evaluation of medical education, including new programmes, is necessary.

Longitudinal Integrated Clerkships (LICs) are one such new programme type within medical education. LICs are placements in which students are sited within a clinical setting ‘or a variety of interlinked clinical settings’ for ‘an extended time’, where continuity in patient interaction, supervision and assessment aid not only cognitive knowledge acquisition but also ‘cultural learning’ [4]. The term ‘Longitudinal Integrated Clerkship’ was first used in relation to the Cambridge Integrated Clerkship at Harvard Medical School in 2007 [5, 6], and the popularity of clerkships identifying as LICs has blossomed since. Prior to 2007, clerkships of this nature existed, such as the Rural Physician Associate Program in Minnesota [7], but were not termed as such. The use of LICs is multifactorial, including use by some institutions as a tool to increase recruitment of practitioners to rural areas [8]. There has been significant recent progression in LIC development and adoption—in 2014, 54 LIC programmes were documented in seven different countries, more than doubling the number of programmes on offer documented in Norris et al.’s 2009 review [9]. Recent figures suggest a sustained appetite for LICs—in 2018, 98 longitudinal programmes were reported in the USA alone, 26 of which met strict international criteria for definition as an LIC [10].

As LICs emerge as an increasingly popular model of clinical education delivery, research regarding their use and impact is increasing. An increasingly detailed literature base necessitates periodic synthesis, in order to best direct future research and avoid duplication of effort. Yet, literature reviews specific to research regarding LIC use are infrequent. More broadly, literature reviews exist that include LIC related literature, such as Crampton et al.’s systematic review of undergraduate placements in underserved areas [11] and Somporn et al.’s narrative review of stakeholder experiences of rural community-based medical education [12]. Although these reviews are clearly of use in contributing to wider discourse within medical education, e.g. regarding education in underserved areas, they do not synthesise research pertinent only the use and impact of LICs. To the author’s best knowledge, only three formal LIC-specific literature reviews have been published—Walters et al.’s narrative review synthesising the outcomes, including academic performance, of LICs for students [13], Brown et al.’s narrative review on the development and implementation of LICs [14] and Bartlett et al.’s narrative review regarding development of a sustainable LIC [15].

A relative dearth of literature reviews pertinent to LICs would not be reason enough to conduct this scoping review. Yet, as LIC research grows, it becomes more difficult to ascertain the contemporary literature base—what is known, what has already been done, what would it be worthwhile asking or doing? Research regarding professional identity formation within LICs has not been synthesised or summarised in this way. Identity is a broad concept that can be explored from a multitude of theoretical perspectives, including theory which conceptualises identity as a cognitive process, sociocultural theories and social constructionist conceptualisations. As there is no consensus definition of professional identity or organising theoretical perspective, identity research can itself be broad and disparate [16]. Within medical education, identity research has occupied a marginalised position within medical education journals, explored instead from a ‘broader health and social sciences arena’ [16]. It remains unclear what the sum of knowledge regarding identity development within Longitudinal Integrated Clerkships is and whether it too struggles with nebulosity and marginalisation. In particular, the theory that has been used to explore identity formation within LICs, and the ways in which identity has been conceptualised in regard to LIC research, has not been summarised. As such, any impact of this new model of educational delivery on identity formation and directions for future novel research regarding identity within LICs remain unclear. This scoping review will map the current landscape of identity development research within LICs, highlighting avenues for future research questions, popular philosophical conceptualisations of identity and theoretical gaps.

## Methods

### Objectives

This scoping review aims to identify and synthesise the literature on professional identity formation within Longitudinal Integrated Clerkships. The PS (Population, Situation) tool [17] has been used to form a research question for this work:

- Population: health professions students
- Situation: identity formation within Longitudinal Integrated Clerkships

The research question of this review is what is known about professional identity formation within Longitudinal Integrated Clerkships?

A scoping review has been selected as an exploratory form of knowledge synthesis. Identity is a broad concept, and as no previous literature reviews focus on identity formation within Longitudinal Integrated Clerkships, it is unclear what is already known. Therefore, a scoping review synthesising current knowledge and mapping areas for future research is necessary.

### Methodological framework

This scoping review is registered with Open Science Framework (registration ID: osf.io/hk83p) and will be reported in accordance with the guidelines provided in the Preferred Reporting Items for Systematic Reviews and Meta-Analyses (PRISMA) extension for scoping reviews (PRISMA-ScR) [18]. This protocol has been reported in accordance with the reporting guidance provided in the PRISMA extension for protocols (PRISMA-P) [19] (see PRISMA-P checklist in additional file 1).

Arksey and O’Malley’s six scoping review steps [20] have been used to guide the methods of this work and will be used as a scoping review framework throughout this process.

### Search and information sources

The following relevant databases will be searched (from inception onwards): MEDLINE, EMBASE, PubMed, Web of Knowledge, ERIC, PsychInfo, Google Scholar, JSTOR, Scopus and Web of Science. The search strategy will include both Medical Subject Headings (MeSH) and free text terms, combined with Boolean operators. No restrictions of date will be placed upon the search.

In addition, the reference lists of included studies will be explored for relevant additional work, and the ‘related articles’ function in PubMed will also be used.

A draft search strategy designed to run in MEDLINE is provided in additional file 2. This search strategy will be appropriately translated to run through the other electronic databases previously listed.

### Inclusion criteria

Studies of any design will be considered for inclusion, with both quantitative and qualitative output, so long as the object of study is professional identity within Longitudinal Integrated Clerkships involving health professions students. Reviews will also be considered for inclusion. Although some contest the inclusion of reviews within scoping reviews, scoping reviews represent a ‘preliminary assessment of potential size and scope of available research literature’ [21]—reviews are part of this research literature, the topics of reviews themselves representing contemporary thinking and interest within a field. As such, we believe the inclusion of relevant reviews will add depth to this study and facilitate the mapping of work done, and interest, in this field.

Ideally, all papers will self-define as having studied a Longitudinal Integrated Clerkship. As a relatively new form of medical education, this is important context and so usually detailed in the title, abstract or full text of the work. When it is unclear from the title and abstract whether an educational programme represents a LIC or not, the full text will be reviewed for inclusion against study inclusion criteria. For the purpose of this review, Longitudinal Integrated Clerkships will be defined in line with Norris et al.’s seminal review of LICs [22]. Norris et al. describe three defining features of an LIC: (1) Medical students ‘participate in the comprehensive care of patients over time’; (2) Students ‘participate in continuing learning relationships with these patients’ clinicians and (3) Students ‘meet the majority of the year’s core clinical competencies, across multiple disciplines simultaneously, through these experiences’ [22]. Norris et al. also stress that for a programme to represent an LIC, it should be the ‘central element of [medical student] clinical education’ for the duration of its run [22]. All included studies will be evaluated against these criteria, to ensure their fit as a true Longitudinal Integrated Clerkship. Where it remains unclear how a programme meets this criteria, corresponding authors will be contacted for further clarification.

### Exclusion criteria

Research involving the study of Longitudinal Integrated Clerkships, or their variants, in postgraduate training (e.g. in the UK foundation programme, internships, residency) will be excluded. Working as a doctor carries a different set of influences on identity development than being a student does [23], and beginning work as a doctor represents an identity transition. Postgraduate medical identity following this transition is, therefore, difficult to compare with pre-qualification identity. Currently, LICs for health profession students are more populous than postgraduate LICs, as is student-based LIC research [8, 24]. As such, only LICs run for health professional students will be eligible for inclusion in this work, to best and most accurately represent the bulk of the LIC literature base.

Practically speaking, articles for which the full text is unavailable following direct contact of the corresponding author will be excluded. Unfortunately, there is no budget in this study for translation, so articles not in English will also be excluded.

There will be no exclusions based on date published; all existing literature will be searched.

### Selection of studies

An automated text-classification programme will be developed using Python and used to process all retrieved search results [25]. All search results will be extracted from the previously listed electronic databases as .csv files. All data will be merged and screened for duplicates using Python. Duplicates will be removed from the data set. Hand-screening results located throughout the study will also be fed into Python, listed as a .csv file.

Initially, deductive coding will be performed. A Python script will be written to generate a frequency count of the different terms used by authors in their titles. Terms found in more than 1% of publications will be reduced to their simplest root form, e.g. ‘identity’, ‘identify’ and ‘identification’ would all become the root ‘identit’, which will then be used as a wildcard. Words found in greater than or equal to 5% of all titles will be arranged in tabular form (tabulated). The list of all tabulated root words obtained will then be fed into another Python programme. The second Python programme will code all titles to produce a binary matrix. In the binary matrix, 0 will signal ‘no match’, and 1 will signal ‘match’.

Titles will be used to generate a frequency count in preference to abstracts, as has been done in previous deductively coded literature searches [26]. The rationale for this decision centres around the fact that authors use titles to signpost the most important contents of their work and so as not to miss articles without abstracts and bias results [26].

The second Python programme will then be used again to inductively analyse publication titles and abstracts. This will act as another layer of screening, ensuring appropriate capture of relevant studies. An extensive list of keywords will be developed using a thesaurus that relate to the focus of this study—professional identity and Longitudinal Integrated Clerkships. These keywords will be fed into the Python script to once again code titles with a frequency count, generating another binary matrix. Once title coding is complete, this process will be re-run using study abstracts, to ensure all relevant articles have been captured. Once completed, all studies identified as a ‘match’ by all generated binary matrices will be manually screened for inclusion against the study eligibility criteria by two authors (MB and PW), working in EndNote. Disagreement will be resolved by initial discussion. If a consensus cannot be reached, a third reviewer (GF) will be consulted for their interpretation. All studies identified for inclusion will undergo data extraction, charting, mapping and thematic content analysis.

### Data extraction and charting

Two authors (MB and PW or GF) will read each article meeting the work’s inclusion criteria in depth. All relevant data will be transferred to a data extraction sheet, which will be iteratively developed by the entire research team. Each author’s data extraction will be independently checked by another member of the research team for accuracy. Version 1 of this data extraction form has been drafted (see additional file 3), but changes to this form are anticipated, in line with inductive development. At this early stage, it is anticipated that relevant data will include demographic information (list of authors, year published, setting/context), methodological detail (study design and any given methodology), the study’s research questions, detail on relevant identity theory used, study conclusions and identified directions for future research.

## Data synthesis

### Quantitative textual meta-analysis

Results of the deductive coding generated by both Python programmes will be presented as a quantitative textual meta-analysis. Word frequency counts will suggest what professional identity research within Longitudinal Integrated Clerkships as a field is most concerned with.

### Qualitative thematic analysis

Thematic analysis will be undertaken on all studies identified that meet this work’s inclusion criteria. Thematic analysis of all identified relevant studies will be presented in narrative format.

## Discussion

This scoping review will explore professional identity formation within Longitudinal Integrated Clerkships, evaluating any known impact of the educational model and mapping the ways in which identity within LICs has been researched. The review will follow standardised, accepted scoping review processes, adhering closely to PRISMA-P guidance [19] and Arksey and O’Malley’s framework [20]. If necessary, protocol amendments will be recorded in the master protocol document, and the reasons for any amendments clearly noted. Outputs reporting the scoping review’s results will also report any such amendments. Combining deductive and inductive coding will allow for a more thorough, systematic evaluation regarding the relevance of identified literature. The code used to create both Python programmes will be made open access by the GitHub repository, to increase transparency and replicability of this work. Inclusion of an independent software developer within the research team, who works regularly with Python programming language, has helped to develop robust deductive coding programmes and ensure methodological rigour.

Although the planned literature search as part of this scoping review is comprehensive and was developed in consultation with an information search specialist, it is anticipated that there will be limitations to this review. Although most practical in our setting, excluding articles that are not written in English is not without issue. There is the potential for valuable non-English language work to be overlooked. Additionally, although significant effort will be made to identify relevant identity studies within the Longitudinal Integrated Clerkship literature base, it is possible that relevant studies prior to definition of the LIC term in 2007 and those that do not list their LIC context within their title and abstract will be missed.

Ultimately, this scoping review will ascertain what is known about professional identity development within Longitudinal Integrated Clerkships, mapping current knowledge using both quantitative and qualitative means. This should highlight whether LICs as an educational model hold any benefit in fostering professional identity development and outline gaps in what is known to date. It may well be that overlap exists in what is known about identity development within LICs and identity development in other educational models (such as the more traditional ‘block rotation’ model), but as the evidence regarding identity development in LICs has not yet been synthesised, this remains unclear. Theory used in the exploration of identity development within LICs will also be mapped, in order to relate what is known to broader identity background literature and summarise the main theoretical orientations of current LIC-specific findings. It is hoped that theoretical mapping will illuminate which theories have been most heavily used and avenues for future theoretical exploration of identity within LICs.

The results of this scoping review will be written up for publication in a peer-reviewed medical education journal. Presentation of findings at appropriate, relevant national and international conferences will also be sought. Knowledge will be shared locally, within the author’s own institution, to inform future medical education research.

## Supplementary information

**Additional file 1:** PRISMA-P 2015 Checklist.

**Additional file 2.**

**Additional file 3:** Data extraction sheet- scoping review.

## Data Availability

Not applicable

## Abbreviations

LIC: Longitudinal Integrated Clerkship
PRISMA: Preferred Reporting Items for Systematic Reviews and Meta-Analyses
PRISMA-P: Preferred Reporting Items for Systematic Reviews and Meta-Analyses Protocols
OSF: Open Science Framework
.CSV file: Comma separated values file
