# Supplementary figures and images for: Professional identity formation within Longitudinal Integrated Clerkships: a scoping review protocol
Source: Syst Rev. 2020 Jul 24;9:166. doi: 10.1186/s13643-020-01422-6 (PMC7382026; doi:10.1186/s13643-020-01422-6)

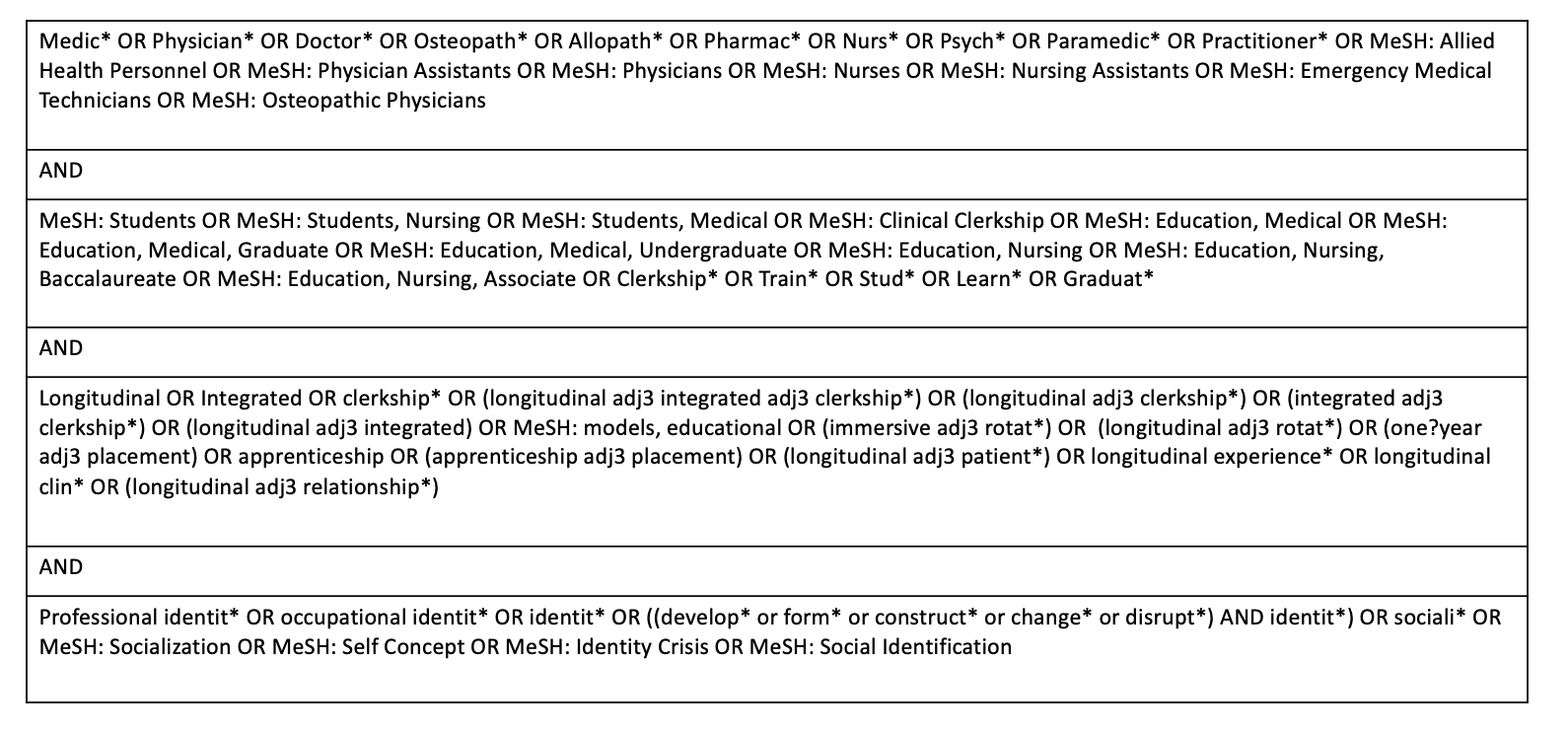

Supplement: Supplementary file 2 — Additional file 2. [file 13643_2020_1422_MOESM2_ESM.png]
