# Supplementary material for: Professional identity formation within Longitudinal Integrated Clerkships: a scoping review protocol
Source: Syst Rev. 2020 Jul 24;9:166. doi: 10.1186/s13643-020-01422-6 (PMC7382026; doi:10.1186/s13643-020-01422-6)
Supplement: Supplementary file 3 — Additional file 3: Data extraction sheet- scoping review. [file 13643_2020_1422_MOESM3_ESM.docx]

**Data extraction sheet- scoping review**

| Authors | Year published | Setting/context | Methods  (study design, methodology) | Research question(s)? | Identity theory used (if any).  How identity is defined, if at all. | Study conclusions (particularly in relation to identity) | Identified directions for future research |
| --- | --- | --- | --- | --- | --- | --- | --- |
|  |  |  |  |  |  |  |  |
|  |  |  |  |  |  |  |  |
